# ETHYLENE RESPONSE FACTOR39–MYB8 complex regulates low-temperature-induced lignification of loquat fruit

**DOI:** 10.1093/jxb/eraa085

**Published:** 2020-02-19

**Authors:** Jing Zhang, Xue-ren Yin, Heng Li, Meng Xu, Meng-xue Zhang, Shao-jia Li, Xiao-fen Liu, Yan-na Shi, Donald Grierson, Kun-song Chen

**Affiliations:** 1 Zhejiang Provincial Key Laboratory of Horticultural Plant Integrative Biology, Zhejiang University, Hangzhou, China; 2 The State Agriculture Ministry Laboratory of Horticultural Plant Growth, Development and Quality Improvement, Zhejiang University, Hangzhou, China; 3 Plant & Crop Sciences Division, School of Biosciences, University of Nottingham, Loughborough, UK; 4 School of Horticulture and Plant Protection, Key Laboratory of Plant Functional Genomics of the Ministry of Education, Yangzhou University, Yangzhou, China; 5 Fondazione Edmund Mach, Italy

**Keywords:** ERF, ERF–MYB complex, lignification, loquat, low temperature, transcriptional regulation

## Abstract

Flesh lignification is a specific chilling response that causes deterioration in the quality of stored red-fleshed loquat fruit (*Eribotrya japonica*) and is one aspect of wider chilling injury. APETALA2/ETHLENE RESPONSIVE FACTOR (AP2/ERF) transcription factors are important regulators of plant low-temperature responses and lignin biosynthesis. In this study, the expression and action of 27 AP2/ERF genes from the red-fleshed loquat cultivar ‘Luoyangqing’ were investigated in order to identify transcription factors regulating low-temperature-induced lignification. *EjERF27*, *EjERF30*, *EjERF36*, and *EjERF39* were significantly induced by storage at 0 °C but inhibited by a low-temperature conditioning treatment (pre-storage at 5 °C for 6 days before storage at 0 °C, which reduces low-temperature-induced lignification), and their transcript levels positively correlated with flesh lignification. A dual-luciferase assay indicated that EjERF39 could transactivate the promoter of the lignin biosynthetic gene *Ej4CL1*, and an electrophoretic mobility shift assay confirmed that EjERF39 recognizes the DRE element in the promoter region of *Ej4CL1.* Furthermore, the combination of EjERF39 and the previously characterized EjMYB8 synergistically transactivated the *Ej4CL1* promoter, and both transcription factors showed expression patterns correlated with lignification in postharvest treatments and red-fleshed ‘Luoyangqing’ and white-fleshed ‘Ninghaibai’ cultivars with different lignification responses. Bimolecular fluorescence complementation and luciferase complementation imaging assays confirmed direct protein–protein interaction between EjERF39 and EjMYB8. These data indicate that EjERF39 is a novel cold-responsive transcriptional activator of *Ej4CL1* that forms a synergistic activator complex with EjMYB8 and contributes to loquat fruit lignification at low temperatures.

## Teaser

EjERF39, a novel cold-responsive AP2/ERF transcription factor, is involved in loquat fruit lignin biosynthesis by interacting with EjMYB8 and directly regulating the phenylpropanoid pathway gene *Ej4CL1*.

## Introduction

Low temperature is one of the major abiotic constraints limiting the quality and yield of crops and horticultural products (Liu *et al.*, 1998). The responses of plants to low temperatures involve a range of physiological and biochemical changes, including plasma membrane rigidification, the accumulation of cryoprotective compounds such as soluble sugars, amino acids, and organic acids, and the activation of some branches of the phenylpropanoid pathway (Dixon and Paiva, 1995; Thomashow, 1999; Chinnusamy *et al.*, 2007; Guy *et al.*, 2008), which sometimes leads to lignification. Lignification is a common symptom that occurs in many chilling-sensitive fruits, such as loquat (Cai *et al.*, 2006*c*), pear (Lu *et al.*, 2015), mangosteen (Dangcham *et al.*, 2008), zucchini (Carvajal *et al.*, 2015), and kiwifruit (Suo *et al*., 2018) when they are subjected to inappropriate low temperature, and this causes a deterioration of quality that severely limits the storage period. Despite the fact that the understanding of low-temperature-induced lignification could improve fruit quality and prolong postharvest storage time, knowledge of the details of the regulatory mechanism of fruit lignification is very limited.

Lignin is a complex phenylpropanoid-derived polymer (Ralph *et al.*, 2004) that contributes to plant secondary cell wall thickening and enhances adaptation to various abiotic stresses, including low temperature (Moura *et al.*, 2010; Ramakrishna and Ravishankar, 2011; Domon *et al.*, 2013; Shafi *et al.*, 2014; Le *et al.*, 2015; Ployet *et al.*, 2018). The plant lignin biosynthesis pathway involves many enzymatic steps, and the enzymes and corresponding genes have been intensively studied in many species (Boerjan *et al.*, 2003; Bonawitz and Chapple, 2010; Shi *et al.*, 2010; Carocha *et al.*, 2015). The expression of genes encoding phenylalanine ammonia lyase (PAL), 4-coumarate:CoA ligase (4CL), cinnamate 3-hydroxylase, hydroxycinnamoyl-CoA shikimate/quinate hydroxycinnamoyl transferase, cinnamoyl-CoA reductase (CCR), and cinnamyl alcohol dehydrogenase (CAD) is dramatically altered by low temperature (El Kayal *et al.*, 2006; Huang *et al.*, 2010; Janska *et al*., 2011; Ployet *et al.*, 2018).

Loquat (*Eriobotrya japonica* Lindl.) is a subtropical fruit of high economic value and can be divided into two groups according to the flesh color (Wang *et al.*, 2010). Red-fleshed cultivars are sensitive to chilling and very likely to exhibit chilling-induced lignification if stored at temperatures of 0–4 °C, and the resulting increase in fruit firmness and lignin content occurs mainly during the first 6 days of storage (Cai *et al*., 2006*b*, *c*). White-fleshed cultivars are more tolerant to low temperature, and lignification and fruit firmness are maintained with only limited changes at low temperature (Wang *et al.*, 2010). Loquat flesh lignification, expressed as an increase in fruit firmness, accumulation of lignin content, and reduction of juice yield, also accompanies other symptoms of chilling injury, such as internal browning (Cai *et al.*, 2006*c*). Due to the significant impact of lignification on fruit quality and marketability, various strategies have been developed to alleviate low-temperature-induced flesh lignification, such as low-temperature conditioning (LTC), heat treatment, and the application of methyl jasmonate and acetylsalicylic acid (Cai *et al*., 2006*a*, *c*; Cao *et al.*, 2008; Zeng *et al.*, 2016). Enzymes within the phenylpropanoid pathway, such as PAL, 4CL, and CAD, show increased activities in response to low temperature, and their transcript levels are correlated with the induction of lignification (Cai *et al.*, 2006*c*; Shan *et al.*, 2008; Li *et al.*, 2017; Xu *et al*., 2014). As has been found in model plants, loquat fruit lignification is also regulated by MYB and NAC transcription factors. Three *MYB* genes, two of the activator type (*EjMYB1* and *EjMYB8*) and one repressor type (*EjMYB2*), have been shown to have transcriptional effects on the promoters of lignin biosynthesis genes, including *EjPAL1*, *Ej4CL1*, and *Ej4CL5*, and influence low-temperature-induced postharvest flesh lignification (Xu *et al.*, 2014; Wang *et al.*, 2016). *EjNAC3*, a transcription factor related to the low-temperature response, regulates lignin biosynthesis by directly binding to the promoter of the *EjCAD-like* gene (Ge *et al.*, 2017).

In addition to these MYB and NAC transcription factors, AP2/ERF members have also been shown to have the potential to regulate genes involved in lignin biosynthesis. For example, overexpression of *AtSHN* (an ERF member) in rice caused an obvious reduction in total lignin content, particularly the G units of lignin composition (Ambavaram *et al.*, 2011); rice *OsERF71*, which was involved in drought resistance, controlled lignin biosynthesis by directly binding to the promoter of *OsCCR1* (Lee *et al.*, 2016); and *Ii049* (a Soloist AP2/ERF member) positively regulated lignin biosynthesis in *Isatis indigotica* (Ma *et al.*, 2017). In loquat, EjAP2-1, a transcriptional repressor, was shown to interact with EjMYB1/2 and inhibited low-temperature-induced lignification (Zeng *et al.*, 2015). Additionally, transcriptomic analysis of postharvest loquat fruit conducted by Liu *et al* (2019), involving comparisons between low-temperature storage, LTC, and heat treatment, identified the most differentially expressed genes in the AP2/ERF family, which may potentially play roles in low-temperature-induced lignification of loquat fruit.

In the present study, 27 *EjAP2/ERF* genes were identified based on RNA-seq data. Their expression in response to low-temperature storage and LTC treatment was analyzed and their potential transcriptional regulatory effects on lignin biosynthesis gene promoters were studied using dual-luciferase and electrophoretic mobility shift assays. The potential interactions between AP2/ERF and transcription factors known from previous work to be involved in low-temperature-induced lignification were also studied.

## Materials and methods

### Plant materials and treatments

Two loquat fruit cultivars were selected for this study, the red-fleshed cultivar ‘Luoyangqing’ (LYQ) and the white-fleshed cultivar ‘Ninghaibai’ (NHB). LYQ undergoes low-temperature-induced lignification whereas NHB does not.

Fruit of uniform size with no visible signs of wounding or disease were selected for postharvest treatments. Commercially mature LYQ loquat fruit were harvested from an orchard at Luqiao (Zhejiang, China) in 2013 and 2015. In 2013, LYQ loquat fruit were stored at two different temperatures (approximately 450 fruit in each batch), one at room temperature (stored at 20 °C for 6 days) and the other at low temperature (0 °C for 6 days). Fruit were sampled at 0, 1, 2, 4, and 6 days of room-temperature and low-temperature storage. For the low-temperature storage experiment conducted in 2015, the fruit were divided into two batches of approximately 450 fruit. The first batch of fruit was kept at 5 °C for 6 days and then transferred to 0 °C (LTC); the second batch was immediately stored at 0 °C for 6 days and used as a control. In the 2015 experiment, fruit were sampled at 0, 0.5, 1, 2, and 6 days of postharvest storage. The white-fleshed NHB fruit were obtained in 2012 from Zhenhai (Zhejiang, China) and stored at 0 °C for 6 days as a postharvest treatment, and fruit were sampled at 0, 1, 2, and 6 d.

All treatments described above were performed with three biological replicates. Fruit flesh (three replicates, four fruit per replicate) without skins and stones were sliced and collected at each sampling point and stored at –80 °C for further use.

### Fruit firmness

Fruit firmness was measured using the TA-XT plus Texture Analyzer (Stable Micro Systems, UK) with a 5 mm diameter probe. After removing a small piece of peel, the fruit flesh was penetrated at a rate of 1 mm s^–1^ to a depth of 4 mm. Firmness measurements of each fruit were taken at two positions 90° apart at the fruit equator and the results were averaged (Wang *et al.* 2010). Fruit firmness was expressed as Newtons (N) and measured from 10 individual fruit replicates at each sampling point.

### Gene isolation and sequence analysis

Sequenced fragments of the unigenes annotated as *AP2/ERF* transcription factors were obtained from the postharvest LYQ loquat fruit RNA-seq database (Liu *et al.*, 2019). Full-length sequences of each gene were isolated using a SMART RACE cDNA amplification Kit (Clontech, CA, USA). The RACE primers are described in Supplementary Tables S1 and S2 at *JXB* online. The sequences of full-length *AP2/ERF* genes were amplified and confirmed with primers listed in Supplementary Table S3 and assembled with the previously reported *EjAP2/ERF* gene data (Zeng *et al.*, 2016) to avoid the inclusion of redundant sequences. Non-redundant loquat and Arabidopsis AP2/ERF sequences were aligned using the neighbor-joining method in ClustalX (v. 1.8.1), and a phylogenetic tree was constructed with Figtree (v.1.3.1).

### RNA extraction and real-time PCR analysis

Total RNA was extracted from frozen loquat fruit flesh samples using the cetyltrimethylammonium bromide method described by Shan *et al.* (2008). Total RNA was treated with a TURBO DNA-free kit (Ambion) to remove genomic DNA, and 1 µg DNA-free RNA was used to synthesize first-strand cDNA with the iScript^TM^ cDNA Synthesis Kit (Bio-Rad).

The gene-specific oligonucleotide primer pairs for real-time PCR were designed with Primer3 (http://primer3.ut.ee/); primers are described in Supplementary Table S4. The reaction mixture (20 µl total volume) for real-time PCR consisted of 10 µl SYBR PCR supermix (Bio-Rad), 6 µl diethylpyrocarbonate-treated H_2_O, 2 µl diluted cDNA template, and 1 µl of each primer (10 µM). Real-time PCR was performed with a Bio-Rad CFX96 instrument (Bio-Rad) using the following PCR procedure: a pre-denaturation step of 95 °C for 30 s, followed by 95 °C for 10 s and 60 °C for 10 s for 45 cycles, completed with a melting curve analysis.

### Dual-luciferase assay

To investigate the transactivation activity of *EjAP2/ERFs* on promoters of loquat lignin biosynthesis genes, dual-luciferase assays were performed according to the protocol described by Yin *et al.* (2010). Full-length *EjAP2/ERF* genes were amplified with primers listed in Supplementary Table S5 and integrated into the pGreenII 0029 62-SK vector. The constructs of fruit lignification-related *EjMYBs* and promoters of lignin biosynthesis genes (*EjPAL1*, *Ej4CL1*/*5*, *EjCAD-like*, *EjCAD3*, and Arabidopsis lignin biosynthetic pathway genes) used for the dual-luciferase assay were originally prepared by Xu *et al.* (2014) and Wang *et al.* (2016).

*Agrobacterium tumefaciens* strain GV3101 transformed with SK- and LUC- constructs were collected and resuspended in infiltration buffer (10 mM MES, 10 mM MgCl_2_, and 0.2 mM acetosyringone) and adjusted to a standard concentration (OD_600_=0.75). The dual-luciferase assays were performed in *Nicotiana benthamiana* leaves with transcription factors and promoters combined in a ratio of 10:1 (v:v). Three days after infiltration, discs from the infiltrated tobacco leaves were collected and the ratio of enzyme activities of firefly luciferase (LUC) and renilla luciferase (REN) was measured. The result obtained with empty vector SK mixed with the promoters was set to a value of 1, as a calibrator. To assess the synergistic effect of two transcription factors on the target promoter, the two transcription factors and promoters were combined in a ratio of 5:5:1 (v:v:v). The effect of each transcription factor with empty vector SK was also tested with the target promoter as a control. Each analysis was carried out with at least three replicates.

### Purification of EjERF39 and electrophoretic mobility shift assay

The full-length cDNA sequence of *EjERF39* without stop codon was inserted into the pGEX-4t-1 (GE) vector and introduced into *Escherichia coli* strain BL21 (Novagen). Expression of the recombinant pGEX-EjERF39 protein in BL21 was fully induced by the addition of IPTG at a final concentration of 1 mM at 16 °C. The target protein was purified following the instructions of the GST-tag Protein Purification Kit (Beyotime), and checked and visualized using SDS-PAGE with Coomassie blue staining. The 40 bp *Ej4CL1* promoter probes containing either the wild-type C-repeat/dehydration-responsive element (DRE) (GCCGAC) or a mutated DRE (AAAAAA) were synthesized and 3′ end labeled with biotin (GeneBio), and cold competitor probes were generated without biotinylation. Details of the electrophoretic mobility shift assay (EMSA) experiments were described by Ge *et al.* (2017).

### Subcellular localization of transcription factors

*Agrobacterium* containing 35S-EjERF39-GFP and 35S-EjMYB8-GFP were resuspended in medium and transiently expressed in leaves of 4-week-old RFP-H2B transgenic *N. benthamiana* plants (Huang *et al.*, 2013). The green fluorescent protein (GFP) and red fluorescent protein (RFP) fusion proteins were examined and imaged 30 h after infiltration using a Zeiss LSM710NLO confocal laser scanning microscope. GFP and RFP fluorescence was detected at laser wavelengths of 488 nm and 543 nm, respectively.

### Bimolecular fluorescence complementation

Bimolecular fluorescence complementation (BiFC) assays were performed to analyze the protein–protein interaction between EjERF39 and EjMYB8 using the protocols described by Li *et al.* (2017) with some modifications. Full-length *EjERF39* and *EjMYB8* sequences, without termination codons, were constructed in vectors containing C-terminal or N-terminal fragments of yellow fluorescent protein (YFP), respectively, and transiently expressed by *Agrobacterium*-mediated infiltration into leaves of 5-week-old *N. benthamiana* plants. Primers used are listed in Supplementary Table S6. The YFP fluorescence of tobacco leaves was imaged 2 days after infiltration using a Zeiss LSM710NLO confocal laser scanning microscope.

### Firefly luciferase complementation imaging

A luciferase complementation imaging (LCI) assay with pCAMBIA-nLUC and pCAMBIA-cLUC vectors was used to validate the protein–protein interaction between EjERF39 and EjMYB8 (Li *et al.*, 2017). The LUC enzyme was divided into the N-terminal (nLUC) and C-terminal (cLUC) portions. EjMYB8 was fused with nLUC in the pCAMBIA-nLUC vector and EjERF39 was fused with cLUC in the pCAMBIA-cLUC vector. Primer pairs used for vector construction are shown in Supplementary Table S7.

*Agrobacterium* strains harboring either nLUC, cLUC, nLUC-EjMYB8, or cLUC-EjERF39 were resuspended in infiltration buffer at a final concentration of OD_600_=0.75. Equal volumes of each strain were mixed and incubated for 3 h at room temperature before infiltration. Luciferase activity was observed 2 days after infiltration with luciferin (0.2 mM) sprayed on to the infiltrated position of the leaves and kept in the dark for 30 min. The experiment was imaged with a NightSHADE LB 985 imaging system (Berthold) and repeated with three to five independent biological replicates.

## Results

### Isolation of loquat *AP2/ERF* genes and phylogenetic analysis

**Fig. 1.**
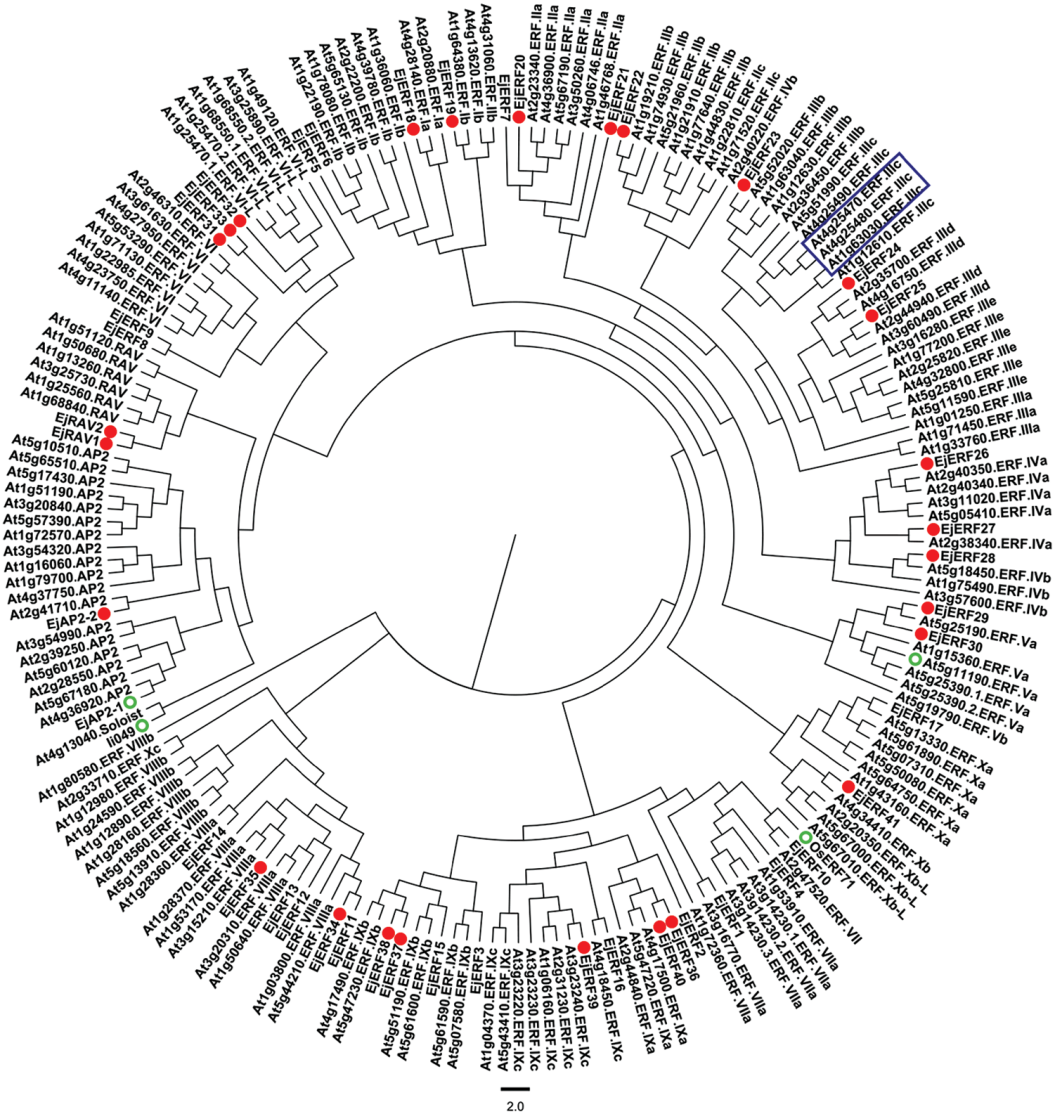
Phylogenetic analysis of *EjAP2/ERF* genes. The deduced amino acid sequences of Arabidopsis *AtAP2/ERF* genes were obtained from NCBI and TAIR. The newly identified loquat *EjAP2/ERF* genes are marked with solid circles and lignin-related *AP2/ERF* genes are marked with open circles. Arabidopsis *DREB1/CBF* members are enclosed with a box.

Twenty-seven *EjAP2/ERF* genes, assigned to either the ERF family (*EjERF18*-*EjERF41*, MH753387-410), AP2 family (*EjAP2-2*, MH753413), or RAV family (*EjRAV1* and *EjRAV2*, MH753411-2), were isolated based on the loquat RNA-seq database (Liu *et al.*, 2019). Phylogenetic analysis indicated that 24 *EjERF* genes were distributed into nine subfamilies, including subfamilies I, II, III, IV, V, VIII, IX, X and VI-L (Fig. 1), according to Nakano *et al.* (2006).

### Relationship between *EjAP2/ERF* expression and low-temperature-induced lignification

**Fig. 2.**
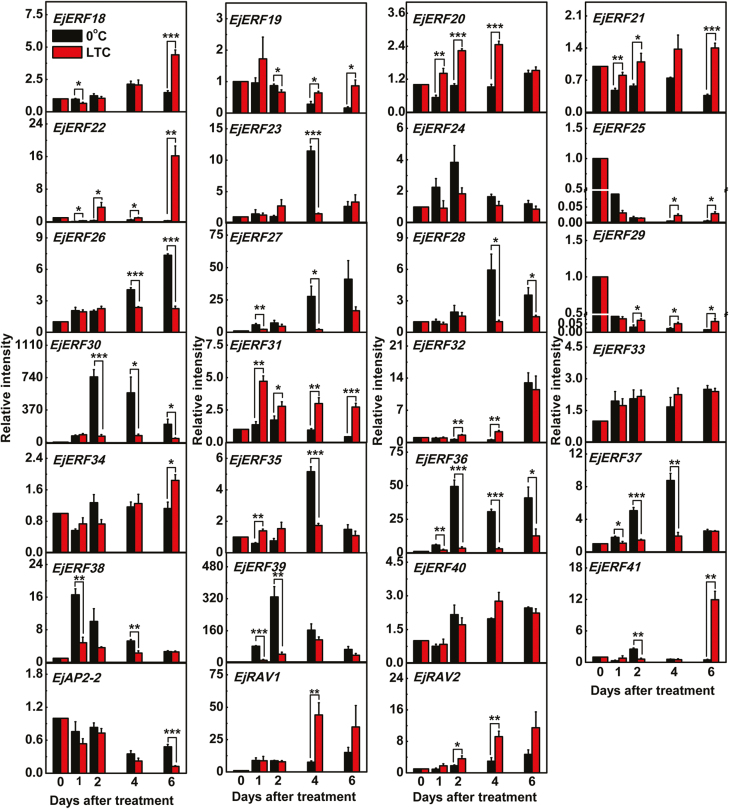
Expression levels of *EjAP2/ERF* genes in response to 0 °C and LTC treatments in LYQ loquat fruit. mRNA levels are presented as a ratio relative to the harvest time point (0 days), the value of which was set as 1. Error bars indicate the SE from three replicates. Statistical analyses were performed using Student’s *t*-test: *P<0.05, **P<0.01, ***P<0.001. The materials were collected and treatments conducted with three biological replicates as described by Zeng *et al.* (2015).

The relationship between the 27 newly isolated *EjAP2/ERF* genes and fruit lignification was evaluated in LYQ loquat fruit using immediate low-temperature storage (0 °C) and LTC treatment. Fruit firmness and lignin content rapidly increased during 0 °C storage, and prior LTC treatment effectively alleviated this process (Xu *et al.*, 2014; Zeng *et al.*, 2015). Transcript analysis indicated that the 27 *EjAP2/ERF* genes responded differentially to 0 °C storage and LTC treatment. *EjERF27*, *EjERF30*, *EjERF36*, and *EjERF39* were most responsive to 0 °C. The transcript abundance of *EjERF27* increased during the whole storage period and reached a maximum abundance of 41-fold relative to day 0 by day 6, while *EjERF30*, *EjERF36*, and *EjERF39* showed greater abundance at day 2 compared with day 0 (748-, 50- and 329-fold increase, respectively) (Fig. 2). The enrichment of transcripts of the same four *EjERF* genes was positively correlated with flesh lignification at 0 °C, and their transcript levels were inhibited in fruit that had previously received LTC treatment (Fig. 2). This reduction in transcript levels by LTC treatment suggested that these transcription factors could play a role in low-temperature-induced flesh lignification, since this is also reduced by LTC.

### Transactivation of promoters of loquat lignin biosynthesis genes by *EjAP2/ERFs*

**Fig. 3.**
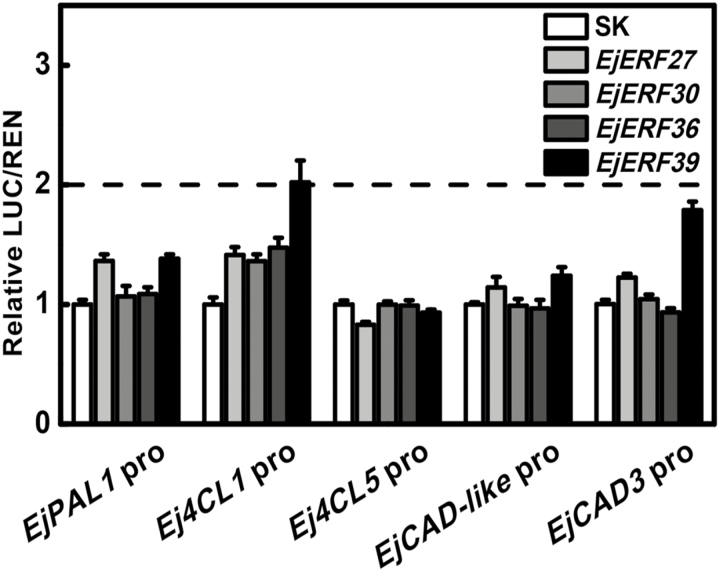
Regulatory effects of *EjAP2/ERF* genes on promoters of loquat lignin biosynthesis genes in loquat identified by a dual-luciferase assay. The LUC/REN ratio of the empty vector (SK) plus promoter was used as a calibrator (value set to 1). Error bars indicate the SE from three replicates.

In order to investigate the possible regulatory role of *EjERF27*, *EjERF30*, *EjERF36*, and *EjERF39* in controlling loquat lignin biosynthesis genes, their activities on the promoters of key genes previously implicated in the control of low-temperature-induced lignification, *EjPAL1*, *Ej4CL1*, *Ej4CL5*, *EjCAD-like*, and *EjCAD3* (Xu *et al.*, 2014; Wang *et al.*, 2016; Ge *et al.*, 2017), were tested. Dual-luciferase assays indicated that EjERF39 could transactivate the *Ej4CL1* promoter, with 2.02-fold enhancement, but had limited or no effect on the other promoters (less than 2-fold increase) (Fig. 3). Despite the correlation between *EjERF27*, *EjERF30*, and *EjERF36* expression and loquat flesh lignification (Fig. 2), these transcription factors had very limited effects on the promoters of any of the loquat lignin biosynthesis genes tested (Fig. 3).

The regulatory effect of *EjERF39* was also tested with promoters of genes involved in the Arabidopsis phenylpropanoid pathway. As shown in Supplementary Fig. S1, *EjERF39* selectively transactivated the promoters of *AtPAL3* and *AtCAD*5 over 2-fold.

### Interaction between *EjERF39* and the *Ej4CL1* promoter

**Fig. 4.**
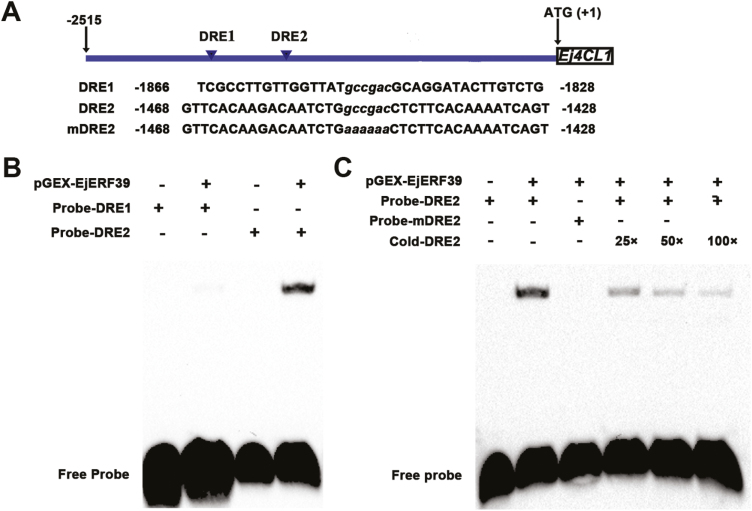
Electrophoretic mobility shift assay (EMSA) of EjERF39 binding to the DRE element in the promoter of *Ej4CL1 in vitro*. (A) DRE elements in the *Ej4CL1* promoter and oligonucleotides used for the EMSA, with the core sequences and base mutant indicated with lower-case letters. (B, C) Biotin-labeled DNA probe from the wild-type promoter or mutant probe was incubated with pGEX-EjERF39 protein. - and + represent the absence or presence of the indicated constructs.

It is well established that AP2/ERF transcription factors preferentially bind to the DRE element of their target gene promoters in response to abiotic stresses such as low temperature (Stockinger *et al.*, 1997). Sequence analysis identified two DRE (GCCGAC) elements in the promoter region of the *Ej4CL1* gene (Fig. 4A), suggesting that it might be the direct target of EjERF39, and EMSA was conducted to validate the interaction of EjERF39 with the *Ej4CL1* promoter. DNA fragments containing the DRE elements (DRE1, DRE2) and a relevant base mutant (mDRE2) were used as probes (Fig. 4A). The results showed that recombinant pGEX-EjERF39 fusion protein could bind one of the DRE elements used as probes (DRE2) but not the other (DRE1), which suggested that EjERF39 physically binds to the promoter of *Ej4CL1* (DRE2 fragment) (Fig. 4B). The specificity of the interaction between EjERF39 and DRE2 but not the DRE1 fragment in the *Ej4CL1* promoter was confirmed by using the mutant probe and cold competitors (Fig. 4C).

### Synergistic effects of EjERF39 and EjMYB8 on *Ej4CL1*

**Fig. 5.**
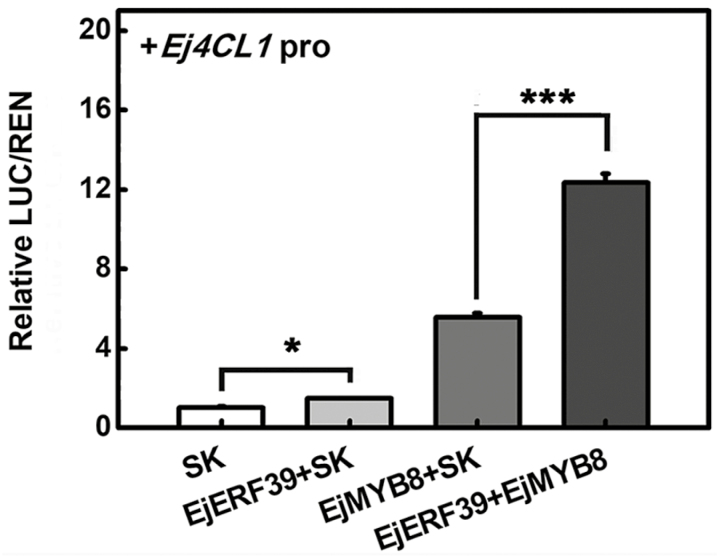
Synergistic transactivation effect of EjERF39 and EjMYB8 on the *Ej4CL1* promoter. The LUC/REN ratio of the empty vector (SK) plus promoter was used as a calibrator (value set to 1). Statistical analyses were performed using Student’s *t*-test: *P<0.05, ***P<0.001.

The results of gene expression and the dual-luciferase assay indicated that EjERF39 could transactivate the promoter of *Ej4CL1* and was a candidate for participation in the cold-induced lignification of loquat fruit. Our previous studies indicated that some other transcription factors were also involved in loquat fruit lignification via direct interaction with the *Ej4CL1* promoter (Xu *et al.*, 2014; Wang *et al.*, 2016). Accordingly, the effects of *EjERF39* and these previously characterized transcription factors were investigated in combination, using the dual-luciferase assay. EjERF39 and EjMYB8 together triggered a substantially higher induction of the *Ej4CL1* promoter, with a LUC/REN ratio of 12.31, compared with transfection of *EjERF39* (1.47-fold activation) or *EjMYB8* (5.55-fold activation) with the empty vector (Fig. 5). The combination of EjERF39 with each of the other transcription factors showed no significant additive effects (Supplementary Fig. S2).

**Fig. 6.**
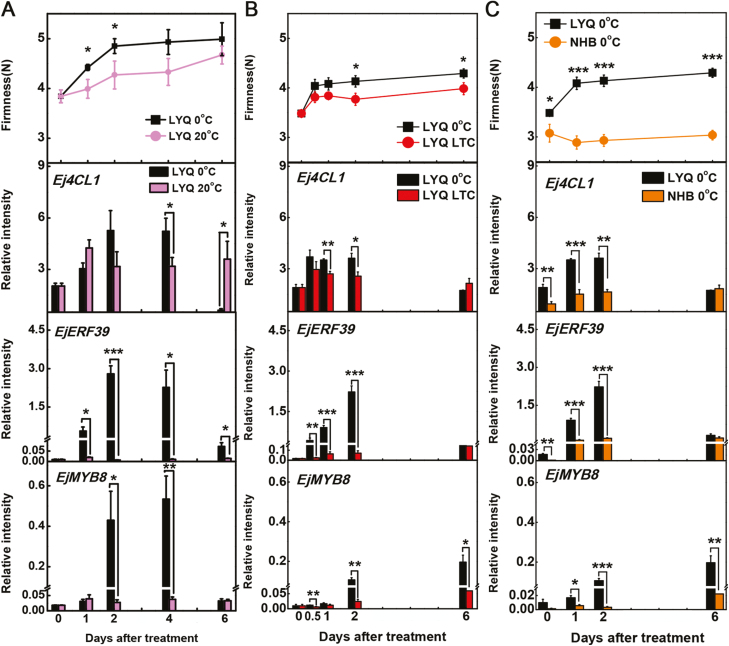
Relationship between firmness (flesh lignification) and the accumulation of *Ej4CL1*, *EjERF39*, and *EjMYB8* transcripts during postharvest treatments of LYQ fruit (which shows low-temperature-induced lignification) (A, B) and in LYQ and the cultivar NHB (which does not show lignification in response to low temperature) (C). Error bars indicate the SE from three replicates. Statistical analyses were performed using Student’s *t*-test: *P<0.05, **P<0.01, ***P<0.001.

The expression of *Ej4CL1*, *EjERF39*, and *EjMYB8* in response to low temperature was measured in different cultivars (Fig. 6). As shown in Fig.6A, *Ej4CL1*, *EjERF39*, and *EjMYB8* were all cold responsive, and fruit exposed to low temperature underwent a corresponding change in firmness. Consistent with the previous results, the expression of *EjERF39* and *EjMYB8* was substantially enhanced by storage at 0 °C and reduced by prior LTC treatment, and the accumulation of *Ej4CL1* transcripts was highly correlated with fruit firmness (Fig. 6B).

The positive correlations between *Ej4CL1*, *EjERF39*, and *EjMYB8* expression and firmness was also examined in fruit of the red-fleshed cultivar LYQ (which is sensitive to low-temperature-induced lignification) and the white-fleshed cultivar NHB (which does not show low-temperature-induced lignification). The firmness of NHB fruit remained unchanged from 3.07 N at day 0 to 3.04 N at day 6 during storage at 0 °C, and there was no obvious enrichment of *Ej4CL1*, *EjERF39*, and *EjMYB8* transcripts in NHB fruit (Fig.6C). The coordinated expression of *Ej4CL1*, *EjERF39*, and *EjMYB8* and the increase in firmness of LYQ fruit undergoing cold-induced lignification, and the absence of any increase in firmness in the low-temperature lignification-free NHB cultivar, suggested that these genes could play vital roles in the low-temperature-induced lignification of red-fleshed loquat fruit.

### Protein–protein interaction between EjERF39 and EjMYB8

**Fig. 7.**
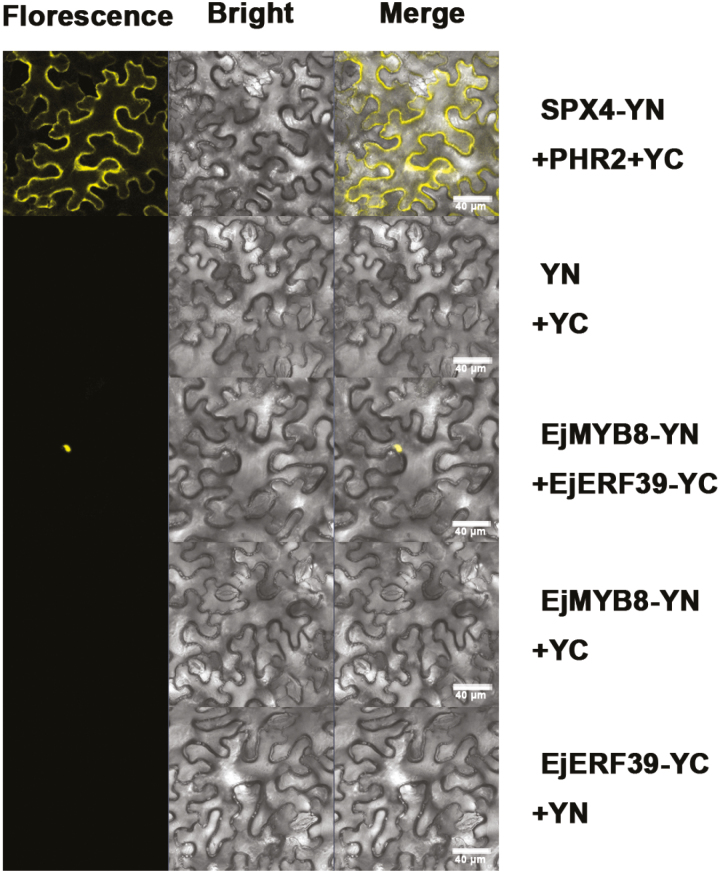
Protein–protein interaction between EjERF39 and EjMYB8 demonstrated using bimolecular fluorescence complementation in tobacco leaves. EjERF39 and EjMYB8 proteins were fused to the C- and N-termini of YFP (YC and YN), respectively. PHR2-YC and SPX4-YN were used as positive controls; YC and YN were used as negative controls. Bars=40 μm.

Subcellular localization of GFP-EjERF39 showed a strong fluorescence signal in the nucleus, while GFP-EjMYB8 may locate to both the nucleus and the cytoplasm or near the plasma membrane (Supplementary Fig. S3). The protein–protein interaction between EjERF39 and EjMYB8 was studied using BiFC. No YFP signal was observed in tobacco leaves expressing the combination of a single construct and the corresponding empty vector or bidirectional empty vectors (negative controls, Fig. 7). Tobacco leaves co-transformed with PHR2-YC and SPX4-YN showed an obvious YFP fluorescent signal in the cytoplasm (positive controls, Fig. 7; Lv *et al.*, 2014). EjERF39-YC co-transformed with EjMYB8-YN also showed a YFP fluorescent signal in the nucleus; these results indicated that EjERF39 could interact in the nucleus with EjMYB8 (Fig. 7).

**Fig. 8.**
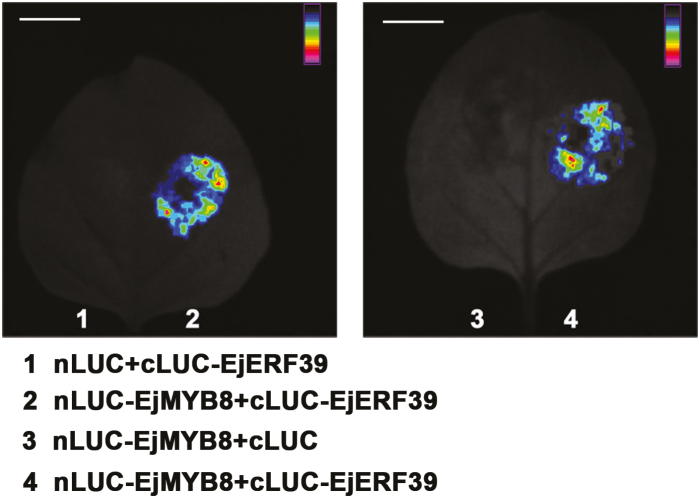
Protein–protein interaction between EjERF39 and EjMYB8 demonstrated using luciferase complementation imaging in tobacco leaves. Bars=5 cm.

In order to verify the results obtained by BiFC, the EjERF39 and EjMYB8 interaction was analyzed by LCI. Co-infiltration of nLUC-EjERF39 and cLUC-EjMYB8 in tobacco leaves led to a strong luminescence signal of LUC (Fig. 8), whereas no obvious LUC activity was detected in negative controls (the combinations of nLUC + cLUC-EjMYB8 or nLUC-EjERF39 + cLUC) (Fig. 8).

## Discussion

Lignification has enabled the long-term adaptation of vascular plants to the terrestrial environment (Campbell and Sederoff, 1996; Bonawitz and Chapple, 2010; Moura *et al.*, 2010). Apart from vegetative tissues, lignin accumulation also occurs in fruit flesh and is widely related to stress conditions such as wounding (Kamdee *et al.*, 2014) and low temperature (Dangcham *et al.*, 2008; Xu *et al.*, 2014), and significantly reduces fruit quality and marketability. Loquat is an ideal fruit for exploring the mechanism of flesh lignification, given the existence of two different types that exhibit distinct low-temperature responses and texture characteristics. Red-fleshed loquat undergoes chilling-induced lignification, while white-fleshed loquat does not (Wang *et al.*, 2010). LTC and heat treatment have been reported to be effective strategies to alleviate low-temperature-induced lignification of red-fleshed loquat (Cai *et al.*, 2006*c*; Zeng *et al.*, 2016). Investigation of the molecular basis for loquat flesh lignification has identified several lignin biosynthesis structural genes (*EjPAL1*, *Ej4CL1*/*5*, *EjCAD-like*, and *EjCAD3*) and transcription factors (*EjMYB1/2/8*, *EjAP2-1*, and *EjNAC3*) involved in low-temperature-induced lignification (Xu *et al.*, 2014; Wang *et al.*, 2016; Zeng *et al.*, 2016; Li *et al.*, 2017). However, compared with model plants, the regulatory mechanism of stress-induced flesh lignification remains unclear.

### Association between *EjAP2/ERF* expression and low-temperature-induced lignification of loquat fruit

*EjAP2-1* was previously reported as a negative regulator of low-temperature-induced lignification in loquat (Zeng *et al.*, 2015). However, the possible roles of other AP2/ERF genes in positively regulating lignification induced by low temperature have not been examined. Accordingly, in this study the relationship between the expression of members of the AP2/ERF family and low-temperature-induced lignification in loquat fruit was analyzed, and four transcription factors (*EjERF27*, *EjERF30*, *EjERF36*, and *EjERF39*) were identified whose expression was positively correlated with lignification induced by low temperature (Fig. 2). LTC, similar to cold acclimation, is an effective method for prolonging storage time and alleviating symptoms of chilling injury for diverse chilling-sensitive fruits, such as grapefruit (Chaudhary *et al.*, 2014), mango (Zhang *et al.*, 2017), peach (Wang *et al.*, 2017), pomegranate (Kashash *et al.*, 2016), and zucchini (Carvajal *et al.*, 2018). LTC can suppress subsequent lignin accumulation during cold storage and reduce the accompanying internal browning of loquat fruit that occurs at low temperature (Cai *et al*., 2006*c*; Tucker *et al.*, 2017). The reduction in expression of *EjERF27*, *EjERF30*, *EjERF36*, and *EjERF39* in response to LTC pretreatment was also correlated with a reduction in firmness (Fig. 2), thus these *EjERF*s may contribute to the lignification process.

The AP2/ERF superfamily plays a pivotal role in various biotic and abiotic stresses, including pathogen infection, wounding, salt, drought, hypoxia, and temperature stress, and responses to several stress-related hormones, such as ethylene, jasmonic acid, and abscisic acid (Mizoi *et al*., 2012; Licausi *et al.*, 2013). For example, ERF-IX subgroup members have been extensively characterized in plant pathogen responses (Lorenzo *et al*., 2002; Moffat *et al.*, 2012), and ERF-VII group genes are involved in the hypoxia and submergence response (Licausi *et al.*, 2010). At low temperatures, members of the *DREB1/CBF* subfamily (subfamily III), *AtDREB1B*/*CBF1*, *AtDREB1C*/*CBF2*, and *AtDREB1A*/*CBF3*, directly bind to DRE elements in the promoter region of cold-responsive related (COR) genes and modulate their expression to enhance chilling/freezing tolerance (Stockinger *et al.*, 1997; Gilmour *et al.*, 1998; Liu *et al.*, 1998; Nakashima *et al.*, 2009). These *DREB*s are major regulators of the cold stress response, and their homologs have been identified in numerous plant species, such as rice (Dubouzet *et al*., 2003), wheat (Vagujfalvi *et al*., 2003), maize (Qin *et al.*, 2004), and tomato (Zhang *et al.*, 2004). Based on phylogenetic analysis, *EjERF27* is a member of subfamily IV, *EjERF30* is a member of subfamily V, and *EjERF36* and *EjERF39* are members of subfamily IX, and thus all belong to clades other than the classical cold-responsive *DREB1/CBF* members (Fig. 1). This finding indicates that *EjERF27*, *EjERF30*, *EjERF36*, and *EjERF39* may be new members of a larger group of cold-responsive genes and candidates for involvement in low-temperature-induced lignification.

### *EjERF39* is a direct activator of lignin biosynthesis via *Ej4CL1* modulation

*Ej4CL1* is an important target of loquat flesh lignification, with its expression being positively correlated with fruit firmness and flesh lignin accumulation (Fig. 6; Li *et al.*, 2017). Transient overexpression of *Ej4CL1* in tobacco leaves significantly induced lignin content (Li *et al.*, 2017). *Ej4CL1* is a direct target of several low-temperature-lignification related transcription factors (*EjMYB1/2/8* and *EjAP2-1*; Xu *et al.*, 2014; Zeng *et al.*, 2015; Wang *et al.*, 2016). Using the dual-luciferase system, we found that *EjERF39* was capable of transactivating the promoter of *Ej4CL1* (with the response being significantly above 2-fold) (Fig. 3), suggesting its potential role in the lignin biosynthesis pathway. The roles of *EjERF27, EjERF30*, and *EjERF36,* were not investigated further. The relationship between *EjERF39* and Arabidopsis lignin biosynthetic structural genes was further tested and the results indicated that *EjERF39* transactivated the promoters of *AtPAL3* and *AtCAD6* in the Arabidopsis lignin biosynthetic pathway (Supplementary Fig. S1). EMSA indicated that EjERF39 could directly bind to one of the DRE elements of the *Ej4CL1* promoter (Fig. 4), which suggested that EjERF39 is a direct activator of the *in vivo* expression of *Ej4CL1*, resulting in fruit lignification.

The observation that EjERF39 preferentially bound only one DRE element rather than both of them is consistent with the behavior of the Arabidopsis homolog *AtERF1* (AT3G23240). *AtERF1* is involved in salt, drought, and heat stress as well as pathogen resistance (Moffat *et al.*, 2012; Cheng *et al.*, 2013). Under different stress conditions, *AtERF1* activates stress-response genes by targeting specific *cis*-elements (GCC boxes during biotic stress and DRE elements during abiotic stress), and *AtERF1* specifically binds to only one of the DRE elements in the promoters of abiotic stress-related genes (e.g. *RD20*, *RD29B*, *COR47*, *HSP101*) (Cheng *et al.*, 2013). *AtERF1* and its homologs belonging to the ERF-IX subgroup have been reported to be involved in regulating several aspects of plant secondary metabolism, such as the biosynthesis of monoterpenoid indole alkaloids (*CrORAC2* and *CrORAC3* in *Catharanthus roseus*; Menke *et al.*, 1999; van der Fits *et al*., 2000, 2001), nicotine alkaloids (seven clustered ERF-IX members are located at the NIC-2 locus in tobacco; Shoji *et al.*, 2010; De Boer *et al.*, 2011), and artemisinin (*AaERF1* and *AaERF2* in *Artemisia annua*; Yu *et al*., 2012). Lignin is also an important secondary metabolite, but the previously reported lignin-related *AP2/ERF* genes, such as *AtSHN* (a member of ERF subfamily V; Ambavaram *et al.*, 2011), *EjAP2-1* (an AP2 member; Zeng *et al.*, 2015), *OsERF71* (a member of ERF subfamily VII; Lee *et al.*, 2016) and *Ii049* (a Soloist member; Ma *et al.*, 2017), belong to different clades from *EjERF39*. All of these data indicate that EjERF39 is a novel regulator of lignification.

### EjERF39 complexes with EjMYB8 and synergistically activates the *Ej4CL1* promoter

Individual transcription factors can be effective regulators of lignin biosynthesis but some also operate by forming complexes with other proteins. For instance, AtMYB75 interacts with AtKNAT7 (a member of the KNOX family) and inhibits lignin synthesis in Arabidopsis stems (Bhargava *et al.*, 2013), and complexes between maize MYB11 and ZML2 (a member of the TIFY family) are involved in wound-induced lignification (Velez-Bermudez *et al*., 2015). In loquat, EjAP2-1 regulates lignin biosynthesis by interacting with MYB (EjMYB1/2; Zeng *et al.*, 2015). However, no such interactions between two transcriptional activators have been investigated in relation to low-temperature-induced lignification.

**Fig. 9.**
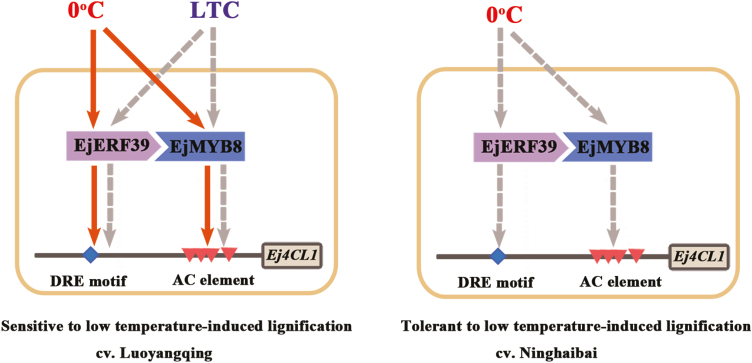
A proposed regulatory model of EjERF39 and EjMYB8 in low-temperature- induced lignification of loquat fruit. Low-temperature (0 °C) storage activates the expression of *EjERF39* (a direct activator of lignin biosynthesis via the DRE motif) and *EjMYB8* (a previously reported lignin-related activator; Wang *et al.* 2016). EjERF39 and EjMYB8, which are involved in the regulation of lignification, can also form protein–protein complexes. Solid arrows indicate significant activations; dashed arrows indicate the absence of activations.

Here, the analysis of synergistic effects in promoter activation and protein–protein interaction studies (Figs 5–8) indicate that EjERF39 is not only a direct regulator of loquat fruit lignification but also interacts with another direct activator, EjMYB8. *EjERF39* was coordinately expressed with *EjMYB8* in response to low temperature, postharvest LTC treatment, and also in different cultivars with distinct texture characteristics (Fig. 6), and the combination of EjERF39 and EjMYB8 greatly enhanced the activation of the *Ej4CL1* promoter (12-fold; Fig. 5). The synergistic effect of EjERF39 and EjMYB8 may be due not only to the fact that they have different *cis*-element targets, with EjERF39 binding to a DRE motif and EjMYB8 binding to the AC element (Fig. 4; Wang *et al.*, 2016) but also to protein–protein interaction between EjERF39 and EjMYB8 (Figs 7 and 8). A proposed model incorporating these findings is shown in Fig. 9. In low-temperature-sensitive LYQ loquat, low temperature (0 °C) up-regulates the expression of *EjERF39* and *EjMYB8*, which are involved in low-temperature-induced lignification by directly activating the promoter of lignification-related *Ej4CL1* and also form protein–protein complexes that lead to flesh lignification and quality deterioration (Fig. 9). LTC treatment blocks the up-regulation of *EjERF39* and *EjMYB8* expression and suppresses low-temperature-induced lignin accumulation (Fig. 9). In the low-temperature-tolerant NHB loquat, expression of *EjERF39* and *EjMYB8* remains low and low-temperature-induced lignification is virtually abolished (Fig. 9). The mechanism underlying the differences in low-temperature response and lignin accumulation between the red-fleshed and white-fleshed cultivars requires further investigation. AP2/ERF–MYB complexes have also been reported to regulate other aspects of fruit quality, including color [PyERF3 and PyMYB114 (Yao *et al.*, 2017), and Pp4ERF24, Pp12ERF96, and PpMYB114 (Ni *et al.*, 2019) in anthocyanin biosynthesis] and volatile compound production (FaERF#9 and FaMYB98 in furaneol synthesis; Zhang *et al.*, 2018). Our results provide a new example of the role of AP2/ERF–MYB complexes in plants and expand our understanding of their roles in plant biology.

In conclusion, the present study has identified 4 AP2/ERF transcription factors that are correlated with low-temperature-induced loquat flesh lignification. *EjERF39* belongs to a different subgroup from the previously reported lignin-related AP2/ERF transcription factors, and acts as a direct activator of the *Ej4CL1* promoter. Moreover, EjERF39 also forms a protein–protein complex with EjMYB8 and enhances the activation of the *Ej4CL1* promoter.

## Supplementary data

Supplementary data are available at *JXB* online.

**Table S1.** Primer sequences for 3′RACE.

**Table S2.** Primer sequences for 5′RACE.

**Table S3.** Primer sequences for full-length amplification.

**Table S4.** Primer sequences for real-time PCR analysis.

**Table S5.** Primer sequences for dual-luciferase assays.

**Table S6.** Primer sequences for BiFC.

**Table S7.** Primer sequences for LCI.

**Fig. S1.** Regulatory effects of *EjERF39* on promoters of Arabidopsis lignin biosynthesis genes using dual-luciferase assay.

**Fig. S2.** Synergistic transactivation effect of EjERF39 and EjMYB1*/2* on the *Ej4CL1* promoter.

**Fig. S3.** Subcellular localization of EjMYB8 and EjERF39.

eraa085_suppl_Supplementary_Figures_S1_S3_Tables_S1_S7

## Abbreviations

AP2/ERF: APETALA2/ETHLENE RESPONSIVE FACTOR
BiFC: bimolecular fluorescence complementation
CAD: cinnamyl alcohol dehydrogenase
CCR: cinnamoyl-CoA reductase
DRE: C-repeat/dehydration-responsive element
EMSA: electrophoretic mobility shift assay
LCI: luciferase complementation imaging
LTC: low-temperature conditioning
PAL: phenylalanine ammonia lyase
4CL: 4-coumarate:CoA ligase.
