# Supplementary material for: ETHYLENE RESPONSE FACTOR39–MYB8 complex regulates low-temperature-induced lignification of loquat fruit
Source: J Exp Bot. 2020 Feb 19;71(10):3172–84. doi: 10.1093/jxb/eraa085 (PMC7475177; doi:10.1093/jxb/eraa085)
Supplement: eraa085_suppl_Supplementary_Figures_S1_S3_Tables_S1_S7 [file eraa085_suppl_supplementary_figures_s1_s3_tables_s1_s7.pdf]

Supplementary figures and tables

ETHYLENE RESPONSE FACTOR EjERF39 complexes with EjMYB8, activate cold-induced lignification of loquat fruit, via the biosynthetic gene *Ej4CL1*

Jing Zhang[1,2,4,#], Xue-ren Yin[1,2,#], Heng Li[1], Meng Xu[1], Meng-xue Zhang[1], Shao-jia Li[1,2], Xiao-fen Liu[1,2], Yan-na Shi[1,2], Donald Grierson[1,3], Kun-song Chen[1,2,*]

Corresponding Author:

Kun-song Chen

Tel: +86-571-88982931

Fax: +86-571-88982224

E-mail: akun@zju.edu.cn

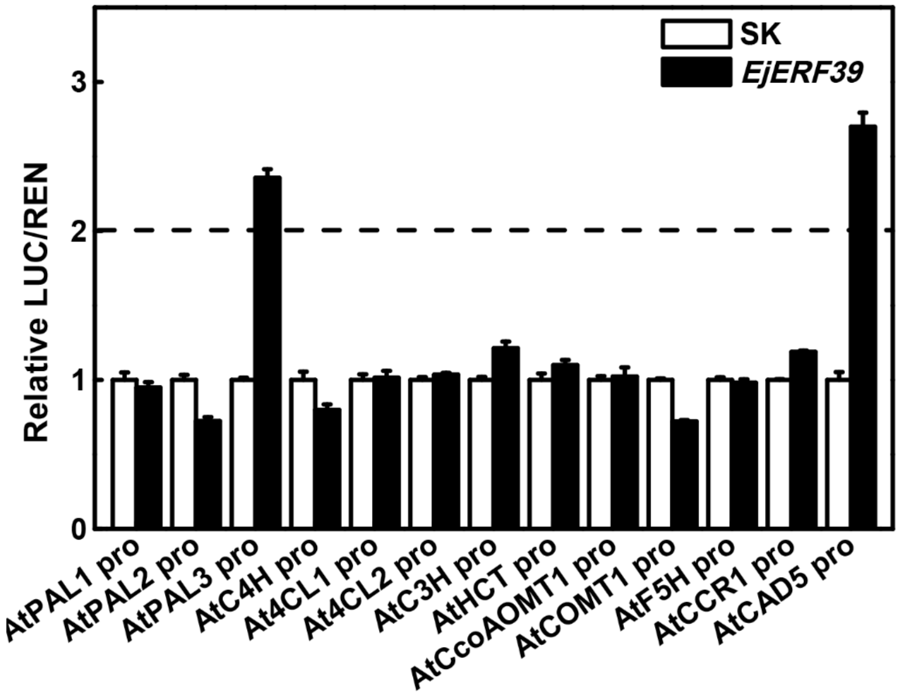

Fig. S1. Regulatory effects of *EjERF39* on promoters of *Arabidopsis* lignin biosynthesis genes using dual luciferase assay.

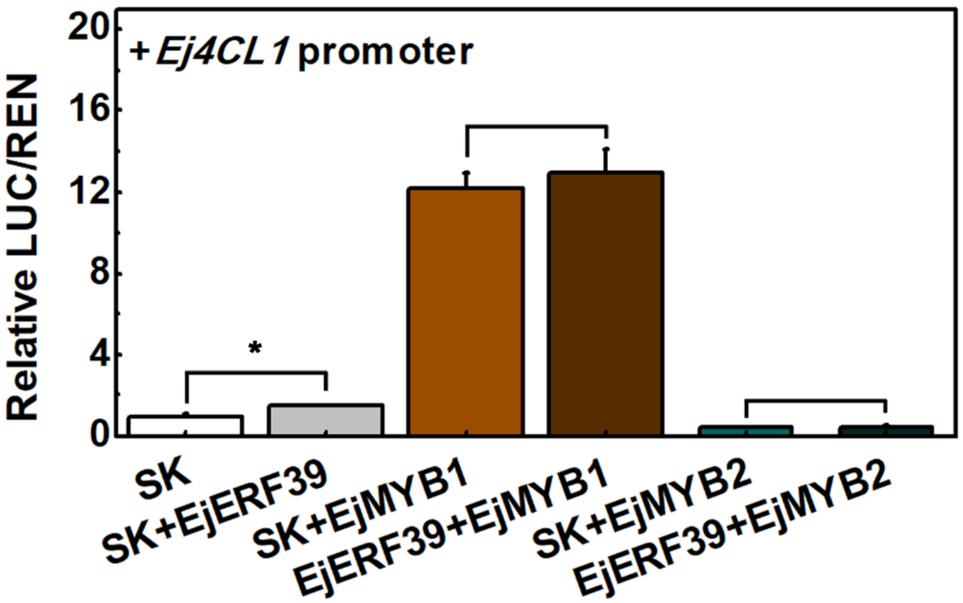

Fig. S2. Synergistic trans-activation effect of EjERF39 and EjMYB1/2 on the Ej4CL1 promoter.

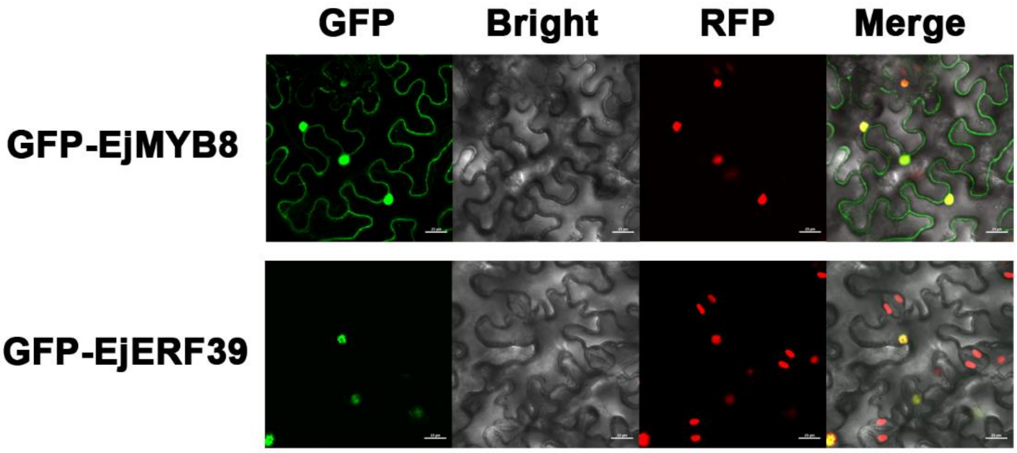

Fig. S3. Subcellular localization of EjMYB8 and EjERF39.

Table S1. Primer sequences for 3'RACE.

Gene	Gene specific primer (5' to 3')	Nested gene specific primer (5' to 3')
EjERF18	GGCGATGATGATGAGCAGATTGGGA	ATGCCGCCGCCACCAGTTCCACATA
EjERF19	GCAGTTCTGTATCCGAGCCGCTGGG	GCCTATAAGCTCCGCGGTGAGTACGC
EjERF20	AAAACCCCTCCTCCCACCTCTCTCT	AGAAGATGGAGTGCGGCGGAGAC
EjERF21	GGGCAAGAACCAAACAATGTTGA	TGATGATCACAGTGGCAGCAACC
EjERF22	TCACTGCATGTCAAACCGCTTCTC	ATACGTCGTCGTCGTCCATGCTC
EjERF23	GGCTTGGCACTTACCCTACGCCGGA	TTCCTCCGCCGCGAGTGACATACGA
EjERF24	GGGGAGTGAGAATGCGTCAATGGGGC	CGCCGCTCTTACCATCAAAGGCCGC
EjERF25	GCACTTCCCCAGCCAGACTCCAAGT	TCATGGATGGGAAAGATGGAAGTGGT
EjERF26	GCGGAGCAGACGAGATGGGAACT	AAAGGCAAGGGAGGGCCTGAGAA
EjERF27	TTGCGGAAGAGGCGCAACAGGTGT	GGGCTCCGTCGAAGGGCTCCAGAAA
EjERF28	TAGAGGAGTGAGGCAGCGGACGTGG	ACCAGCACCCGTCGTCCTCATCGTT
EjERF29#		
EjERF30	ATATCGAGGCGTCCGACAAAGGCAC	CGGGTGCGAAAATGCCGGATATCAG
EjERF31	ACAATGATCCTGATGCCACCGACT	TGCAGGGACCAATGGAGGCAAAA
EjERF32	GATTCGACAAATCTTGGAGAGCTACA	AAGAGATAAACTTGGGACCGGAGCT
EjERF33	TGCCATCGATCACCATCATCTGTGC	GCTGCGCTTTGTTTTGGACTCGAAAGGT
*EjERF34**		
EjERF35#		
EjERF36	GGGGAAGCATTACAGGGGCGTACGG	GGGAGCAGGGATTGGGCAGCAACAA
EjERF37	ACCCAAACCCCAAGTAATCGACCTC	AAACCGCTCCTAAACCCGCCAAC
EjERF38	CCAGGTCCCAATCCGAGTCGTTTTGCT	CGGCGCTCACAATCTCGCTCCCAAG
EjERF39	CTACAGAGGGGTTCGGAGGCGGC	TGTGAGGATAGAGAATGTGGTGGTGT
EjERF40	TGCGAAATGTACGGACAGAGTGGAG	GACTCGATTCGGCGACACTTTCTG
EjERF41	CGCCACTTACTCTCCCCGATGTACT	CCACCGCCAGCAGCTAACTCTCA
EjRAV1	CGAAAGCACACGTACAATGACGAACT G	CTGAACCGCCTCGTGATCCCGAAGC
EjRAV2	GCCAGCAGCGTGATTCTCGACCCC	GCCCTCATCCGCCGAACCCATTTCC
EjAP2-2#		

* *EjERF34* is partial CDS

EjERF29 EjERF35 EjAP2-2 full length transcriptome sequence

Table S2. Primer sequences for 5'RACE

Gene	Gene specific primer (5' to 3')	Nested gene specific primer (5' to 3')
EjERF18	CCCCAAACAATTTCCTCTTGCTGCG	TTGGGTATGTGGAACTGGTGGCGGC
EjERF19&		
EjERF20	GGAGAGAGAGAGAGAGGTGGGAGGA	GCTCTCTTCCTGTTCGCTGTGTCTC
*EjERF21**		
EjERF22	GTGCGACCACGTAAGCAAAAGAGTG	AGACTGAGACTCCACCTGCAACTCG
EjERF23	CCCACCTCCCCACCAACACCCTCAT	GAATCTCGGACACCCATTTCCTGCT
EjERF24	GCCCCATTGACGCATTCTCACTCCCC	CCTCTGCTGCCTCCCATCACCATTA
EjERF25	ACCACTTCCATCTTTCCCATCCATGA	ACTTGGAGTCTGGCTGGGGAAGTGC
*EjERF26**		
EjERF27	CGCCACCCATTTTCCCCAAGTCCTC	TCCGGCCCTCCTTTCCCTCTCATGC
EjERF28	GAACCACCGCCACTCCTGCCACAAC	GGGTGCTGGTGGTGGTCTGGCAAGT
EjERF29#		
EjERF30&		
*EjERF31**		
EjERF32	GAGCTCCGGTCCCAAGTTTATCTCT	AGCAGACGTCTCCAAATCAAGCACT
EjERF33	ACCTTTCGAGTCCAAAACAAAGCGC	AGCACAGATGATGGTGATCGATGGCA
EjERF34	CGTCTTCTCTCTCGGCGCCATAAC	GGTTTCCGTTACCGTTCGCCCTAA
EjERF35#		
EjERF36&		
EjERF37	CTCCATTGGTTTTGGGGGAAGCTG	CGGGTTTAGGAGCGGTTTGGTTCA
EjERF38		
EjERF39&		
EjERF40	GGTCAAACACGGGTACAGGCTACTG	GTAGAGCACCATGTCTTCGGAATCG
EjERF41	GGTGAGGGGAGGAGCGAATTCAAG	ACCCTCTTCTTCGGCCCCTTTTTG
EjRAV1	GGGGTCACGGCTTTCTCGAACAGCT	TCCAGTTCGTCATTGTACGTGTGCT
EjRAV2&		
EjAP2-2#		

* *EjERF21 EjERF26 EjERF31* are partial CDS

& *EjERF19 EjERF30 EjERF36 EjERF39 EjRAV2* full length after 3'RACE

EjERF29 EjERF35 EjAP2-2 full length transcriptome sequence

Table S3. Primer sequences for full-length amplification

Gene	Forward primer (5' to 3')	Reverse primer (5' to 3')
EjERF18	ATGGCTGCAGAAAAAAAGAG	TCAATCTTGGTCCTTCCAAAAG
EjERF19	ATGGACACAATGCAAGAAAACAAT	CTAATTAGCAAGAACTTCCCAAAT
EjERF20	ATGGAGTGCGGCGGAGA	CTAATTCCACTCACCTTCC
*EjERF21**		
EjERF22	ATGTCAAACCGCTTCTCCAA	CTAGAAATTCCAGAGGAAAGAC
EjERF23	ATGGCCGATCCGTATAGC	TTAATAGTTCCAAAGATTTTC
EjERF24	ATGGAAGAAGAACAATTCAATTAT	TCACGCCCACAAGAACTCC
EjERF25	ATGGCAAACCAAATCAACATAG	CTAATACTCCCATAAGAATTG
*EjERF26**		
EjERF27	ATGGAGTCCGAGGCTTCGGATG	TCATTTAGGCAGCCCCTGC
EjERF28	ATGGAGAAGGAAGAGAGAAAG	TTAGTTCCATGATGGAGGTTC
EjERF29	ATGGCTAGATCCCAGCAGCGGTATC	CTATGCAGAAACACCAGGACAAATC
EjERF30	ATGGCGAGACCGCAACAGCGAT	TTAGGTAGCTTGTGGTGGCACA
*EjERF31**		
EjERF32	ATGCCTGAGCCTCGGAGACAGC	TTAATCCAACCAGCAAGCAATGTC
EjERF33	ATGCCGGGGATGAAAACCAT	TCATGGCTTACCATGTTCCA
*EjERF34**		
EjERF35	ATGGCTCCGACGACGGC	TTAGGCGACTTCCGAGGG
EjERF36	ATGCACGGACAGAGCGGAACCGAC	TCAGCTAACCAATAACTGCTCGCC
EjERF37	ATGGCTTTAGATCAAGTCTCG	TTATACGACCATTACTTGTGG
EjERF38	ATGGCTTTAGATCAGGTATCGG	TCATACGACCATAAGCTGTGGAC
EjERF39	ATGCATTGCCACAGGCACACAAAC	TCACCAATTAGGAGTGGCATTAGT
EjERF40	ATGTACGGACAGAGTGGAG	TCAGACCACCAATAACTGCT
EjERF41	ATGCAATATCCCTACAAGCG	TCAAATACTATGAGCATTCCC
EjRAV1	ATGGACGGAATAAGCAGCACAG	CTACAAAGCTCCAATGATCCTT
EjRAV2	ATGGACGGAATAAGCAGCACAG	CTACAAAGCCCCAATGATCCTTG
EjAP2-2	ATGGCGTCGTGGTCCTCGGATCC	TCATTCTTCAGGCTGAAATAAAGTG

* *EjERF21 EjERF26 EjERF31 EjERF34* are partial CDS

Table S4. Primer sequences for real-time PCR analysis

Gene	Forward primer (5' to 3')	Reverse primer (5' to 3')
EjERF18	ATTTCCCTCATCCCTTCTCACTC	CGGTGGTGTGTGTGATAACTAAA
EjERF19	AACAGAGTTTCAAATGGGGATGG	TCAACTCAGGATCAAATGAAGGC
EjERF20	AAAATTGAGGGGTGGGTTTT	AAAGAGATGCAGTAGGCGAAA
EjERF21	CTGACGTCCCAGCATCATC	TCGCGTGGATCTTCTTTTTC
EjERF22	CCGGGATTTGATGAAATGAA	CTTCCGCGAAGTAATCAACG
EjERF23	GGGCTCACGATTTGTATCATCAT	CGTGAATCATACCCTTAATCACTGA
EjERF24	TGATCCTGGGTTTTGCTTCTCTG	GCCAATTAAGCCGCTGATTTCTT
EjERF25	TGACCTACCAGATCTTTTCATGG	CAGTCCTTAATCTGTACTGGCTA
EjERF26	CCATGACTTCGCTGTAAGCA	CCTGTGCCAAAGCTCAAAAT
EjERF27	GGCGTGGATTTCGATTCAGT	GGGGTTAAGGATGCAGCTAAAAT
EjERF28	TATGATGGAGTTGGGAGGAGATG	GACATATTTGACCCTTCGCAAAC
EjERF29	TTGAGCAGATGATTGAGGAGCTT	ACAAGATGCTGAAATGCTAGCTC
EjERF30	GCTGCATTACGGTTCCTTTGA	CGAGATTTTCACGGTTCCTTCT
EjERF31	CAGCGGGTGTTTGAGGTTAT	CATGAGAGTTGGAAAAATCTTGG
EjERF32	AGAGATTAAGACGGTGATTTGCG	CCTTCTGCGACAACAAATCAGAT
EjERF33	AGAGTTCTTGGGTTTTCACTGTG	AGGAGTACACAAAGGCTCAGTAT
EjERF34	GGTCTGGCTCGGAACCTT	CGAAACGAGAGGGAAGTTTG
EjERF35	GTCCTCGGTCGTCTACTTCG	CCGGAAAGAAAGAAAAGGGGT
EjERF36	GGGAAAAGCACAAGTAGCTAACC	CACCCACAAAAGCATCAGATCAG
EjERF37	AAGAAGCGGAAGCTGTGGT	TCACGTCCATTAATTTGTACGG
EjERF38	CGGTGGTGGCTGTAAAGAAG	GGATGAGGAGATAACGGCGA
EjERF39	CAAGTACTAATGCCACTCCTAATTG	CGTAACCCTTCATCTGTTTGCAA
EjERF40	CCAGTAGCACGACTGCTCAA	TCTCGCAAAATAAAAAGCTGAA
EjERF41	GGAAAAGATTGGGGAAGAGG	TCCCCGAATCTGAGGAATTA
EjRAV1	TCGGCAAAAGAATGAGAGAGATG	TACACAACTCACCTTTCTTGCAG
EjRAV2	GGAGTTACTGGAATTAGAGTTTGGC	GCAGCTTCACAAAATGCTAGAGA
EjAP2-2	GGATTACATGCTACAGACACAGG	ATATTCTACACCCCGTATGCAGT

Table S5. Primer sequences used for dual-luciferase assays

Gene	Forward primer (5' to 3')	Reverse primer (5' to 3')
EjERF30-SK	GTGGCGGCCGCATGGCTAGATCCCAGCAGC	TCCACTAGTCTATGCAGAAACACCAGGAC
EjERF36-SK	GTGGCGGCCGCATGCACGGACAGAGCGGAAC	TCCACTAGTTCAGCTAACCAATAACTGCT
EjERF39-SK	GTGGCGGCCGCATGCATTGCCACAGGCACAC	TCCACTAGTTCACCAATTAGGAGTGGCAT

Table S6. Primer sequences for BiFC

Gene	Forward primer (5’ to 3’)	Reverse primer (5’ to 3’)
EjMYB8-YFP^{N}	CGCTTAATTAACATGGTTAGAGCTCCTTGC	GATGGCGCGCCCAATTCCTAGCAATTCTGG
EjERF39-YFP^{C}	CGCTTAATTAACATGCATTGCCACAGGCAC	GATGGCGCGCCCCCAATTAGGAGTGGCATT

Table S7. Primer sequences for LCI

Gene	Forward primer (5' to 3')	Reverse primer (5' to 3')
EjMYB8-nLUC	GAGCTCGGTACCATGGTTAG AGCTCCTTGCTGCC	GATCTGGTCGACAATTCCTAGCA ATTCTGGAATTTC
EjERF39-cLUC	CGGGGCGGTACCATGCATTG CCACAGGCACACAA	CTGCAGGTCGACTCACCAATTAG GAGTGGCATTAGTA
